# Effect of Frankincense Extract on Nerve Recovery in the Rat Sciatic Nerve Damage Model

**DOI:** 10.1155/2016/3617216

**Published:** 2016-04-10

**Authors:** Xiaowen Jiang, Jun Ma, Qingwei Wei, Xinxin Feng, Lu Qiao, Lin Liu, Binqing Zhang, Wenhui Yu

**Affiliations:** Department of Veterinary Medicine, Northeast Agricultural University, 59 Mucai Street, Xiangfang District, Harbin 150030, China

## Abstract

This study investigated the effect of frankincense extract on peripheral nerve regeneration in a crush injury rat model. Forty-eight Sprague-Dawley rats were randomly divided into four groups: control and frankincense extract low-, medium-, and high-dose groups. At days 7, 14, 21, and 28 following the surgery, nerve regeneration and functional recovery were evaluated using the sciatic functional index (SFI), expression of GAP-43, and the proliferation of Schwann cells (SCs) in vivo and in vitro. At day 7, the SFI in the frankincense extract high-dose group was significantly improved compared with the control group. After day 14, SFI was significantly improved in the medium- and high-dose groups. There was no significant difference in GAP-43 expression among the groups at day 7. However, after day 14, expression of GAP-43 in the high-dose group was higher than that in the control group. Histological evaluation showed that the injured nerve of frankincense extract high-dose group recovered better than the other groups 28 days after surgery. Further, S100 immunohistochemical staining, MTT colorimetry, and flow cytometry assays all showed that frankincense extract could promote the proliferation of SCs. In conclusion, frankincense extract is able to promote sciatic nerve regeneration and improve the function of a crushed sciatic nerve. This study provides a new direction for the repair of peripheral nerve injury.

## 1. Introduction

Posttraumatic peripheral nerve repair is a major challenge in restorative medicine. Peripheral nerve injuries may result in temporary or life-long neuronal dysfunction that can lead to economic or social disability [1, 2]. In recent years, pharmacologic agents and immune system modulators have been investigated to enhance nerve regeneration [3–9]. Most treatments for peripheral nerve injury achieve recovery to a great extent in animal models [10] but there are few effective clinical drug treatments available. The most common types of peripheral axon injuries are lacerations, as well as stretch and compression injuries [2]. Following peripheral nerve injury, Wallerian degeneration occurs, a process of acute myelin and axonal degeneration in the distal area of the damaged nerve. This process is in connection with macrophage infiltration, Schwan cell proliferation, and axonal regrowth [11].

Traditional Chinese medicine has a long history and rich experience in the treatment of peripheral nerve injury. Fewer side effects and its effectiveness for multiple targets have led to the increasing importance of traditional Chinese medicine for promoting peripheral nerve regeneration [12–14]. Frankincense is a fragrant gum from trees of the genus* Boswellia* (family Burseraceae) and is produced primarily from several varieties found in Somalia, Yemen, and Oman. It is commonly used to reduce swelling and alleviate the pain caused by inflammatory diseases or tumors and to invigorate blood circulation [15, 16]. Moreover, the boswellic acids isolated from frankincense have potential immunomodulatory effects [17, 18].

In the present study, we evaluated the effects of frankincense on peripheral nerve regeneration using an established rat sciatic nerve injury model. We investigated functional and histologic changes following a sciatic nerve crush injury. The relative expression of GAP-43 was investigated as well as the level of SCs. The present study provides a theoretical basis for the development of natural drugs for the treatment of peripheral nerve injury.

## 2. Materials and Methods

### 2.1. Animals and Grouping

A total of 48 healthy adult male Sprague-Dawley rats with body weights of 200–250 g (purchased from Harbin Medical University) were used in this study. The rats were maintained under specific pathogen-free laboratory conditions on a 12 h light/dark cycle with free access to food and water. The rats were randomly divided into four groups: control (*n* = 12), frankincense extract high-dose (*n* = 12), frankincense extract medium-dose (*n* = 12), and frankincense extract low-dose (*n* = 12).

### 2.2. Animal Models

The rats were anesthetized with an intraperitoneal injection of 10% chloral hydrate (3 mL/kg) and then shaved and washed with antiseptic solution before positioning for surgery. Surgery was performed by clamping the left sciatic nerve 3 times for 10 s each using 2 mm wide pincers. The distal injury was marked with a 10-0 nylon microscopic suture in the epineurium. Complete crush was confirmed by the presence of a translucent band across the nerve. The incision was then closed in layers (muscle and skin) with absorbable sutures. All operations were performed on the left hindlimb, and the right limb served as a nonoperated control. All surgeries were completed by one person under aseptic conditions.

### 2.3. Drug-Delivery Method and Dose

The rats were given frankincense extract (purchased from Xi'an Xu-Huang Bio-Tech Co., Ltd.) via intragastric administration on the first day after surgery: high-dose group: 300 mg/kg; medium-dose group: 150 mg/kg; low-dose group: 75 mg/kg. The control group was given an equivalent volume of distilled water.

### 2.4. Observed Index and Methods

#### 2.4.1. Sciatic Functional Index

Evaluation of the sciatic functional index (SFI) was performed on days 7, 14, 21, and 28 following surgery. The rats were put on a confined walking track (10 cm × 50 cm) with a dark shelter at one end. White office paper was placed on the bottom of the track. The rat's hindlimbs were dipped in water-soluble blue ink, and the rat was allowed to walk down the track, leaving its hind footprints on the paper. The following measurements were taken from the footprints: (1) the print length; (2) the toe spread; and (3) the intermediary toe spread. Using these data, the SFI was calculated using the following formula derived by Bain et al. [19] (E: sciatic nerve crush side; N: normal side); functional recovery was assessed by calculating the SFI ratio with the SFI value of 0 set as normal and the SFI value of −100 set as complete injury:

(1) SFI=109.5ETS−NTSNTS−38.3EPL−NPLNPL+13.3EIT−NITNIT−8.8.

#### 2.4.2. The Expression of GAP-43 in the Injured Nerve

A sample of crushed nerve (about 1 cm) was excised on days 7, 14, 21, and 28 following surgery. Samples containing 30 *μ*g of total protein from the injured nerve were subjected to 12% SDS-PAGE, electroblotted onto PVDF membranes (Biosharp), and then blocked with 5% nonfat milk for 2 h at 20°C. The membranes with the transferred proteins were incubated with the rabbit anti-GAP-43 primary antibody (1 : 400, Beijing Biosynthesis Biotechnology Co., Ltd.), followed by incubation with horseradish peroxidase-conjugated goat anti-rabbit immunoglobulin G (1 : 1,000) as the secondary antibody. Chemiluminescence reaction was carried out with an ECL kit (Biosharp) for 1 min, followed by exposure to a Kodak X-Omat radiographic film. Similar procedures were carried out with an anti-*β*-actin antibody (1 : 1,000; Beijing ZSGB-Biotechnology Co., Ltd.). Relative expression was calculated as the ratio of the gray value of the target protein band to an internal reference band.

#### 2.4.3. Immunohistochemical Analysis

Immunohistochemical analysis of the sciatic nerves was performed on day 28. The distal segment of sciatic nerve was removed and fixed in a 4% paraformaldehyde solution for 48 h, conventionally dehydrated, cleared, and embedded in paraffin 4 *μ*m thick. Then, dewaxed paraffin sections were treated with 3% hydrogen peroxide for 20 min to block endogenous peroxidases. After three washes, the slices were repaired for 8 min in 0.01 M citrate buffer (pH 6.0) in a microwave. The sections were blocked with bovine serum albumin (BSA) for 20 min at room temperature and incubated at 4°C overnight with anti-S100B (1 : 200, Beijing Biosynthesis Biotechnology Co., Ltd.). The following day, the slices were washed with phosphate-buffered saline and then a secondary antibody labeled with biotin (Beijing ZSGB-Biotechnology Co., Ltd.) was added. Streptavidin labeled with horseradish peroxidase was added and slices were incubated at room temperature for 20 min. The antigens were visualized with 3,3′-diaminobenzidine. The sections were then redyed with hematoxylin for 20 s and fixed with neutral balsam. Sections were processed with Image-Pro Plus 6.0 software.

#### 2.4.4. Hematoxylin-Eosin Staining

At 28 days after surgery, the sciatic nerve at the distal anastomotic site was removed and fixed in 10% neutral formalin for 24 hours, dehydrated through a graded alcohol series, embedded in paraffin, and cut into 4 *μ*m transverse sections. The sections were dewaxed with xylene, hydrated through a graded alcohol series, stained with hematoxylin, treated with acidic alcohol, immersed in running water, stained with eosin, dehydrated, permeabilized, and mounted. The tissue was viewed under a light microscope to examine myelin degeneration and regeneration.

#### 2.4.5. Effect of Frankincense Extract on SCs Proliferation

SCs at the 5th passage (purchased from Chinese Academy of Sciences, Shanghai, China) were used for this experiment. Cells were randomly divided into experimental and control groups. Concentrations of frankincense extract in the culture medium of the experimental groups were 62.5, 125, 250, 500, 1000, and 2000 mg/L. Medium of the control group contained no frankincense extract. SCs (180 *μ*L of 1 × 10^6^/mL) of every group were placed in a 96-well culture plate with DMEM containing 10% fetal bovine serum. Then, 20 *μ*L MTT (5 mg/mL) was added to each well for 4 h. The liquid was removed and 200 *μ*L of dimethyl sulfoxide was added on an oscillator for 10 min. The absorbance of each pore was detected at the wavelength of 490 nm in an enzyme-linked immunosorbent assay meter.

#### 2.4.6. Effect of Frankincense Extract on the Cell Cycle of SCs

The same experimental groups as above were used. Medium containing frankincense extract was added to the experimental group and normal DMEM to the control group. After culturing for 48 h, SCs of every group were harvested, washed with phosphate-buffered saline, and fixed in 70% ethanol overnight. Cells were stained with propidium iodide (1 mL/10^6^ cells) and incubated for 30 min at 37°C. The cell cycle of SCs was detected by flow cytometry and analyzed using Modfit LT software.

## 3. Results

### 3.1. SFI

**Figure 1:**
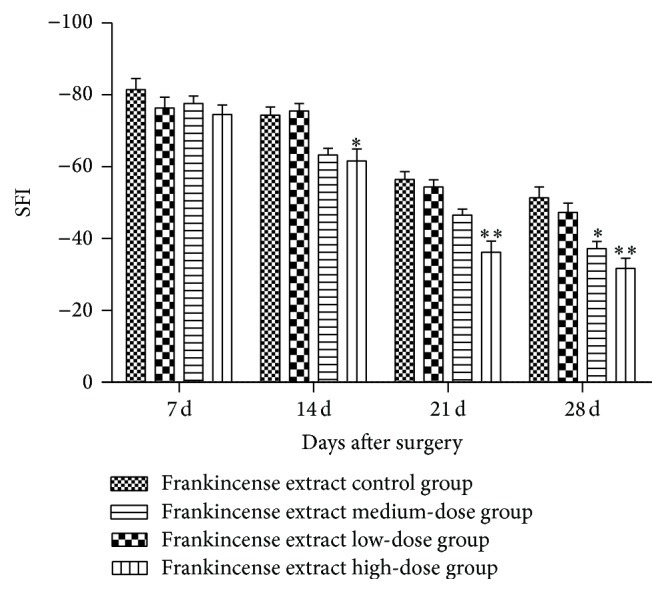
Effects of frankincense extract on the sciatic function index 7, 14, 21, and 28 days after surgery. ^*∗*^Significant difference compared with the control, *p* < 0.05. ^*∗∗*^Significant difference compared with the control, *p* < 0.01.

The effect of frankincense extract on the SFI was calculated on days 7, 14, 21, and 28 after sciatic nerve crush injury. All the groups showed a gradual recovery of sciatic function, but the sciatic function was worse in the control group (Figure 1). At days 14 and 21, comparing to the control group, the neurological function in the frankincense extract high-dose group was significantly improved (*p* < 0.05). At day 28, the neurological function was significantly improved in the medium-dose group and high-dose group compared with the control group (*p* < 0.05). But there was no obvious difference in the low-dose group at any time point.

### 3.2. The Expression of GAP-43

**Figure 2:**
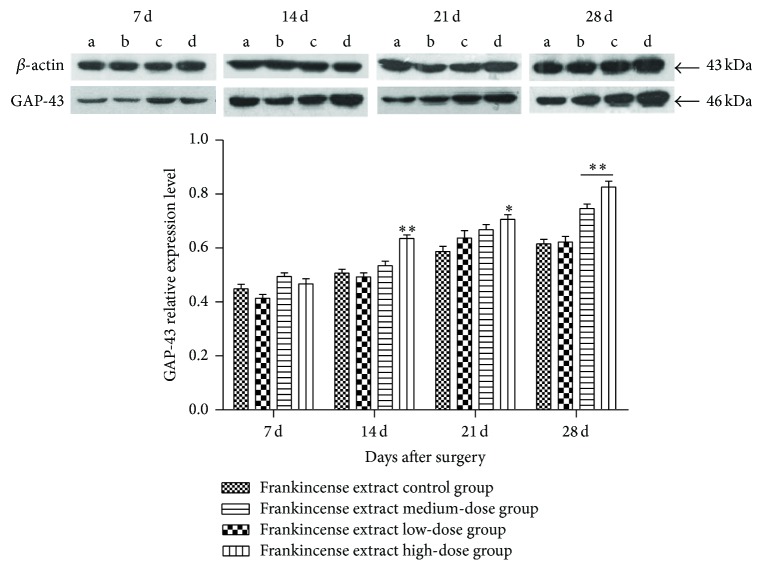
Effects of frankincense extract on GAP-43 relative expression level 7, 14, 21, and 28 days after surgery. (a) Frankincense extract control group. (b) Frankincense extract low-dose group. (c) Frankincense extract medium-dose group. (d) Frankincense extract high-dose group ^*∗*^Significant difference compared with the control, *p* < 0.05. ^*∗∗*^Significant difference compared with the control, *p* < 0.01.

As shown in Figure 2, there was no obvious difference in the low-dose group compared with the control group. But at days 14, 21, and 28, the expression of GAP-43 in frankincense extract high-dose group was significantly higher than control group (*p* < 0.05). At day 28 comparing to the control group, GAP-43 expression was much higher in frankincense extract medium-dose group (*p* < 0.01).

### 3.3. Immunohistochemical Analysis

**Figure 3:**
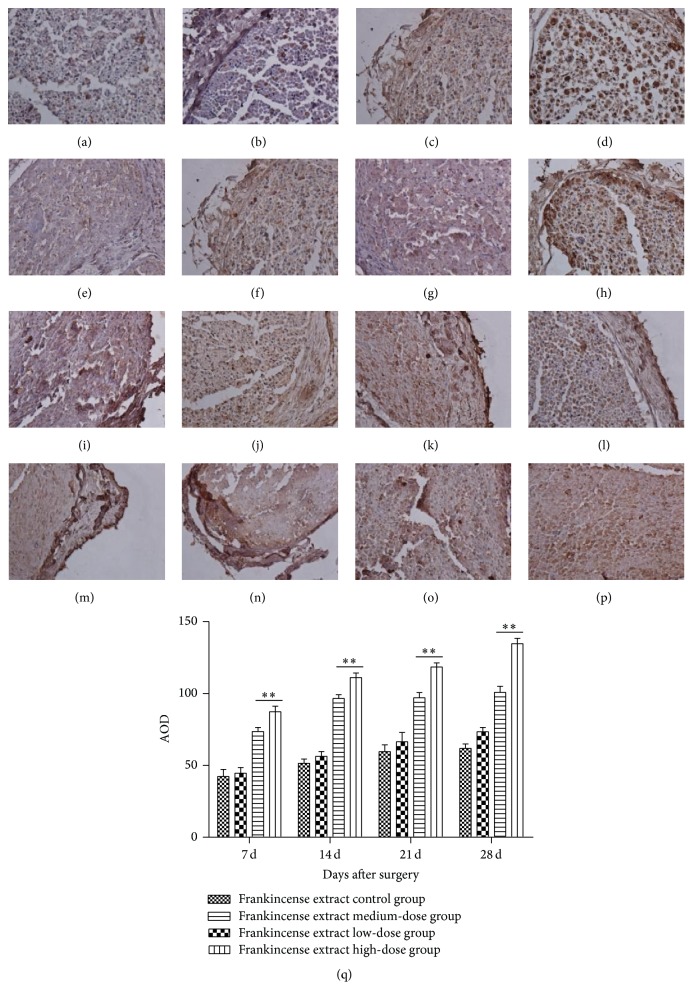
S100 expression in rat sciatic nerve (magnification 400x). (a) Control group, 7 d. (b–d) Frankincense extract low-, medium-, and high-dose groups, 7 d. (e) Control group, 14 d. (f–h) Frankincense extract low-, medium-, and high-dose groups, 14 d. (i) Control group, 21 d (j–l). Frankincense extract low-, medium-, and high-dose groups, 21 d. (m) Control group, 28 d. (n–p) Frankincense extract low-, medium-, and high-dose groups, 28 d. (q) S100-positive cells in normal and crushed nerves. ^*∗∗*^Significant difference compared with the control, *p* < 0.01.

Expression of S100 in frankincense extract medium- and high-dose groups was significantly higher than that in the control group at all time points, but there was no obvious difference in the low-dose group (Figure 3).

### 3.4. Histological Changes in Injured Sciatic Nerve

**Figure 4:**
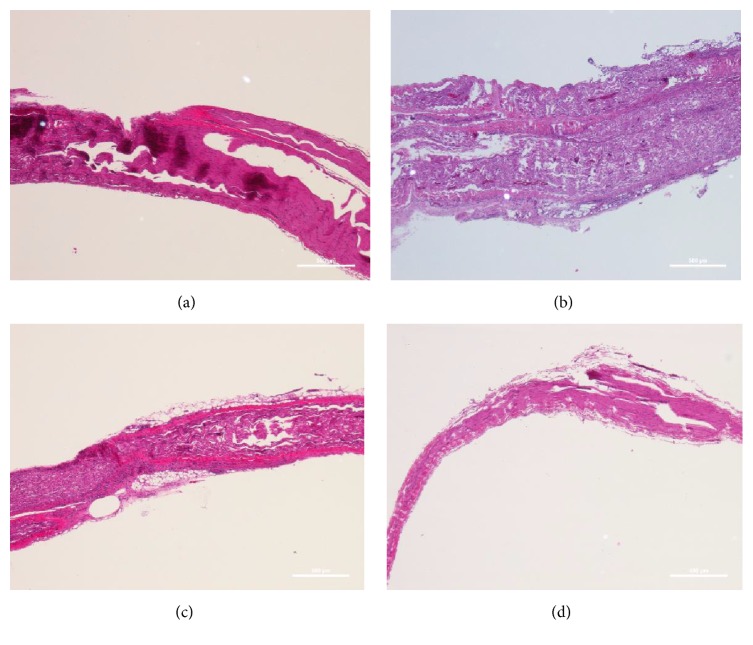
Histological changes in injured sciatic nerve (magnification 40x). (a) Control group. (b) Frankincense extract low-dose group. (c) Frankincense extract medium-dose group. (d) Frankincense extract high-dose group.

In the control group, 28 days after surgery, the nerve fiber at injury site was reconnecting. There was distinct Wallerian degeneration and myelin sheath disintegration. Vacuoles were present in nerve fibers and spindle cells proliferated. In frankincense extract low-dose group, the nerve fiber at injury site was reconnecting, the adjacent axon degenerated, myelin sheath collapsed, vacuoles were seen in nerve fibers, and SCs proliferated. No significant differences were observed in the histology of the injured sciatic nerve between the low-dose group and medium-dose group. But in high-dose group, 28 days after surgery, the injured nerve recovered better than the other groups and SCs proliferated obviously (Figure 4).

### 3.5. Effect of Frankincense Extract on SCs

Less than 48 h after frankincense extract treatment, frankincense extract could promote the proliferation of SCs in a concentration range of 62.5–2000 mg/L (Table 1). Compared with the control group, there was a significant difference in concentrations ranging from 125 to 2000 mg/L. Seventy-two hours after treatment, frankincense extract could promote the proliferation of SCs in concentrations of 125 and 250 mg/L. The result indicates that it could promote the proliferation of SCs less than 48 h after frankincense extract treatment.

### 3.6. Effect of Frankincense Extract on the Cell Cycle of SCs

**Figure 5:**
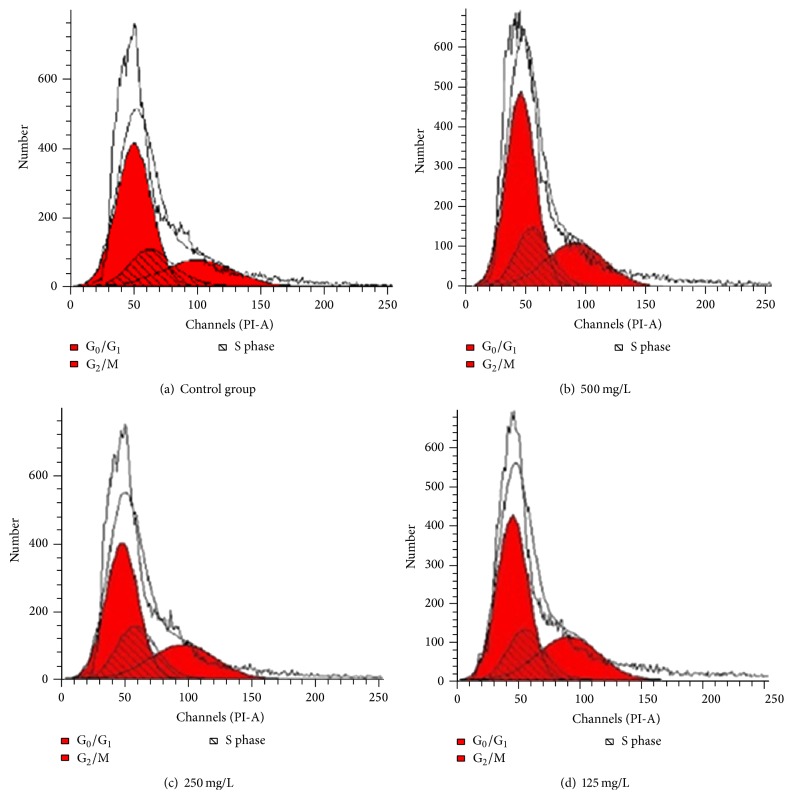
Effect of frankincense extract on the cell cycle of SCs 48 h after frankincense extract treatment; Schwann cells were stained with PI for DNA content analysis by FACS. Details of the experiments are given in Section 2.

The percentage of SCs treated by frankincense extract in the S phase and G_2_/M phase was significantly higher than in the control group (Figure 5, Table 2).

## 4. Discussion

The sciatic nerve crush injury model is a relatively mild nerve injury that is constantly used in studies of nerve regeneration [20–22]. The SFI is one standard for evaluating motor function [23] and it can reflect nerve function after peripheral nerve injury. In our study, all groups showed a gradual recovery of sciatic function. At day 7, SFI in the frankincense extract high-dose group was significantly improved compared with the control group. At days 14, 21, and 28, the neurological function was significantly improved in the medium- and high-dose groups compared with the control group. However, there was no significant difference between the low-dose group and the control group at all time points. These results suggest that frankincense extract can accelerate functional recovery following injury in a dose-dependent manner. What is more, histological evaluation showed, 28 days after surgery, that the injured nerve of frankincense extract high-dose group recovered better than the other groups obviously.

GAP-43 was originally found in neurons and its expression is particularly high during axonal growth, and during development and regeneration in both the central and peripheral nervous systems [24]. During the course of neuronal development or regeneration, the expression of GAP-43 varies over a 100-fold range, from very low levels in resting neurons to high levels in cells undergoing axogenesis or synaptic remodeling [24, 25]. In the present study, we found that high-dose frankincense extract treatment effectively elevated the expression of GAP-43 in the crushed nerve on days 14, 21, and 28 after surgery compared with distilled water treatment. Thus, we postulate that frankincense extract promotes the regeneration and functional recovery of the injured sciatic nerves by upregulating GAP-43 expression.

SCs are glial cells in the peripheral nervous system. They provide a permissive environment for nerve regeneration and express multiple types of neurotrophic factors, such as nerve growth factor and brain-derived neurotrophic factor, to provide trophic support for axon regeneration [26, 27]. S100 exists only in glial cells of the central nervous system and SCs of the peripheral nervous system. We observed the expression of S100 by immunocytochemical staining of the injured nerve, which reflected the proliferation of SCs. As a result, the expression of S100 in all the groups was higher than the control group, inferring that frankincense extract promotes the proliferation of SCs. To further study the effect of frankincense extract on SCs, we treated SCs with frankincense extract of different concentrations in vitro. Frankincense extract of 125, 250, and 500 mg/L promoted the proliferation of SCs significantly and elevated the percentage of cells in S phase and G_2_/M phase. In SCs, G_0_/G_1_ phase cells were decreased and S phase and G_2_/M phase cells increased compared with control group. Frankincense extract promotes the growth and the transition from G_0_/G_1_ phase to S phase in SCs. It can be inferred that the mechanism of how did frankincense extract promote proliferation of SCs was that frankincense extract promotes the growth and the transition from G_0_/G_1_ phase to S phase in SCs. It can shorten the growth retardation, stimulate the cell to enter the active S phase from the stationary G0 phase, and increase the number in G_2_/M cells, leading to the proliferation of cells.

## 5. Conclusions

Taken together, these findings suggest that frankincense extract may promote the regeneration of crushed sciatic nerve by improving the sciatic nerve function and increasing the expression of GAP-43 as well as promoting the proliferation of SCs. However, the exact mechanism has not been clearly defined. Further studies are needed to evaluate the significance of frankincense extract in peripheral nerve regeneration.

## Figures and Tables

**Table 1:** Concentration and time effect of frankincense extract on SCs (*n* = 6, $\overset{-}{x}\pm s$).

Group	Concentration	The proliferation of SCs (*D*_490 nm_)				
	mg/L	12 h	24 h	48 h	72 h	96 h
Control	—	0.8096 ± 0.0153	1.2562 ± 0.0214	1.5700 ± 0.0871	1.4502 ± 0.0101	1.4065 ± 0.0120
	62.5	0.8135 ± 0.0807	1.2412 ± 0.0495	1.5670 ± 0.0143	1.4587 ± 0.0272	1.3944 ± 0.0241

Frankincense extract	125	1.2715 ± 0.0532^*∗∗*^	1.8880 ± 0.0409^*∗∗*^	1.9113 ± 0.0426^*∗∗*^	1.875 ± 0.0167^*∗*^	1.6510 ± 0.0231^*∗*^
	250	1.1008 ± 0.0168^*∗∗*^	1.8567 ± 0.0457^*∗∗*^	1.8493 ± 0.0162^*∗∗*^	1.6367 ± 0.0138^*∗*^	1.4860 ± 0.0431^*∗*^
	500	1.0690 ± 0.0937^*∗∗*^	1.7092 ± 0.0457^*∗∗*^	1.7483 ± 0.0129^*∗∗*^	1.4758 ± 0.0103	1.4199 ± 0.0279
	1000	0.9242 ± 0.07023^*∗*^	1.5838 ± 0.0341^*∗*^	1.6005 ± 0.0117^*∗*^	1.4487 ± 0.0196	1.4013 ± 0.0512
	2000	1.0583 ± 0.1015^*∗*^	1.5833 ± 0.0782^*∗*^	1.5915 ± 0.0782^*∗*^	1.4087 ± 0.0196	1.4013 ± 0.0512

^*∗*^Significant difference compared with the control, *p* < 0.05. ^*∗∗*^Significant difference compared with the control, *p* < 0.01.

**Table 2:** Effect of frankincense extract on the cell cycle of SCs (*n* = 6, $\overset{-}{x}\pm s$, %).

Group	48 h		
mg/L	G_0_/G_1_ phase	S phase	G_2_/M phase
Control	64.45 ± 3.34	18.26 ± 3.41	17.29 ± 2.68
125	55.01 ± 4.12	19.88 ± 1.38^*∗*^	25.11 ± 1.99^*∗*^
250	52.53 ± 2.63	21.26 ± 2.6^*∗*^	26.21 ± 3.09^*∗*^
500	48.67 ± 2.79	23.66 ± 1.38^*∗∗*^	27.67 ± 3.53^*∗∗*^

^*∗*^Significant difference compared with the control, *p* < 0.05. ^*∗∗*^Significant difference compared with the control, *p* < 0.01.
